# A novel human Cdh1 mutation impairs anaphase promoting complex/cyclosome activity resulting in microcephaly, psychomotor retardation, and epilepsy

**DOI:** 10.1111/jnc.14828

**Published:** 2019-08-22

**Authors:** Cristina Rodríguez, Irene Sánchez‐Morán, Sara Álvarez, Pilar Tirado, Daniel M. Fernández‐Mayoralas, Beatriz Calleja‐Pérez, Ángeles Almeida, Alberto Fernández‐Jaén

**Affiliations:** ^1^ Instituto de Investigación Biomédica de Salamanca, Hospital Universitario de Salamanca, CSIC Universidad de Salamanca Salamanca Spain; ^2^ Instituto de Biología Funcional y Genómica, CSIC Universidad de Salamanca Salamanca Spain; ^3^ Genómica y Medicina NIMGenetics Madrid Spain; ^4^ Departamento de Neuropediatría Hospital Universitario La Paz Madrid Spain; ^5^ Departamento de Neurología Infantil, Hospital Universitario Quirónsalud Universidad Europea de Madrid Madrid Spain; ^6^ Centro de Salud Doctor Cirajas Servicio de Atención Primaria de Salud Madrid Spain

**Keywords:** Cdh1, *Fzr1*, microcephaly, mutation, psychomotor retardation

## Abstract

The Fizzy‐related protein 1 (*Fzr1*) gene encodes Cdh1 protein, a coactivator of the E3 ubiquitin ligase anaphase‐promoting complex/cyclosome (APC/C). Previously, we found that genetic ablation of *Fzr1* promotes the death of neural progenitor cells leading to neurogenesis impairment and microcephaly in mouse. To ascertain the possible translation of these findings in humans, we searched for mutations in the *Fzr1* gene in 390 whole exomes sequenced *in trio* in individuals showing neurodevelopmental disorders compatible with a genetic origin. We found a novel missense (p.Asp187Gly) *Fzr1* gene mutation (c.560A>G) in a heterozygous state in a 4‐year‐old boy, born from non‐consanguineous Spanish parents, who presents with severe antenatal microcephaly, psychomotor retardation, and refractory epilepsy. Cdh1 protein levels in leucocytes isolated from the patient were significantly lower than those found in his parents. Expression of the Asp187Gly mutant form of Cdh1 in human embryonic kidney 293T cells produced less Cdh1 protein and APC/C activity, resulting in altered cell cycle distribution when compared with cells expressing wild‐type Cdh1. Furthermore, ectopic expression of the Asp187Gly mutant form of Cdh1 in cortical progenitor cells in primary culture failed to abolish the enlargement of the replicative phase caused by knockout of endogenous Cdh1. These results indicate that the loss of function of APC/C‐Cdh1 caused by Cdh1 Asp187Gly mutation is a new cause of prenatal microcephaly, psychomotor retardation, and severe epilepsy.

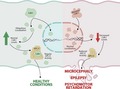

https://doi.org/10.1111/jnc.14835.

Cover Image for this issue: doi: 10.1111/jnc.14524.

## Graphical abstract

We previously found that the anaphase promoting complex/cyclosome (APC/C) inactivation, by deletion of its activator Cdh1, promotes microcephaly in mouse. Here, we describe a *de novo* Cdh1 mutation (p.Asp187Gly) in a 4‐year‐old boy with prenatal microcephaly, psychomotor retardation, and epilepsy. Functional studies in the patient leukocytes, in a human cell line and in mouse neural precursor cells reveal that the Asp187Gly mutation results in decreased Cdh1 protein abundance, likely due to nuclear degradation, leading to APC/C inactivation. Thus, Cdh1 Asp187Gly mutation is herein identified as a novel cause of APC/C‐Cdh1 inactivation triggering prenatal microcephaly, psychomotor retardation, and severe epilepsy in human.

Open Science: This manuscript was awarded with the Open Materials Badge

For more information see: https://cos.io/our-services/open-science-badges/

**Abbreviations used**
APC/C: anaphase promoting complex/cyclosome
BrdU: bromodeoxyuridine
EEG: electroencephalogram
*Fzr1*: Fizzy‐related protein 1
GAPDH: glyceraldehyde‐3‐phosphate dehydrogenase
GFP: green fluorescent protein
HA: hemagglutinin
HEK293T cells: human embryonic kidney 293T cells
OFD: occipitofrontal diameter
RRID: Research Resource Identifier (see scicrunch.org
RT‐qPCR: quantitative reverse transcription‐polymerase chain reaction

The Fizzy‐related protein 1 (*Fzr1*) gene encodes the activator of the E3 ubiquitin ligase anaphase‐promoting complex/cyclosome (APC/C), Cdh1, which regulates mitotic (Watson *et al.* 2019) and non‐mitotic (Almeida 2012; Kimata 2019) functions through interactions with proteins that target for ubiquitination and proteasomal degradation. In late mitosis, APC/C‐Cdh1 activation controls the mitotic exit and G1/G0 progression, thus regulating the onset of DNA replication (Peters 2006; Watson *et al.* 2019). Beyond cell cycle regulation, APC/C‐Cdh1‐linked postmitotic roles have been described (Almeida 2012; Kimata 2019). Components of the APC/C‐Cdh1 complex are ubiquitously expressed in post‐mitotic neurons (Gieffers *et al.* 1999), where it plays a key role in neuronal survival (Almeida *et al.* 2005; Bobo‐Jimenez *et al.* 2017) and function, including axonal growth (Konishi *et al.* 2004), synaptic size and plasticity (van Roessel *et al.* 2004; Teng and Tang 2005; Fu *et al.* 2011; Huang *et al.* 2015) and bioenergetics and antioxidant status of neurons (Herrero‐Mendez *et al.* 2009). Moreover, Cdh1 deficiency impairs learning and memory in the adult brain (Li *et al.* 2008; Bobo‐Jimenez *et al.* 2017). APC/C‐Cdh1 also plays a key role in the developing brain, whereas it regulates neuronal differentiation (Almeida *et al.* 2005; Cuende *et al.* 2008) and neurogenesis (Delgado‐Esteban *et al.* 2013). Functional APC/C‐Cdh1 activity is essential for coupling the progenitor cell cycle exit with the onset of neuronal differentiation during cortex development. However, the impact of APC/C‐Cdh1 in neurological and neurodevelopmental disorders remains largely unknown.

Primary microcephaly refers to a congenitally neurodevelopmental disorder characterized by prenatal reduced cerebral cortex and non‐progressive intellectual disability (Duerinckx and Abramowicz 2018). The correct brain development requires a delicate spatiotemporal balance between the symmetric divisions that maintain the progenitor cell pool, and the asymmetric divisions that generate newly differentiated neurons (Gotz and Huttner 2005). Then, mutations in cell cycle‐related genes have been described to cause microcephaly (Duerinckx and Abramowicz 2018). We previously found that Cdh1 loss causes replicative stress leading to p53‐mediated death of neural progenitor cells and microcephaly (Delgado‐Esteban *et al.* 2013). We now identified a novel missense mutation (c.560A>G) in the human *Fzr1* gene, that predicts an aspartate‐to‐glycine substitution (p.Asp187Gly), which results in severe psychomotor retardation, microcephaly, and refractory epilepsy. Moreover, leucocytes from patient blood showed lower Cdh1 protein levels, but not mRNA expression, in comparison to parental leukocytes. Expression of wild‐type and Asp187Gly mutant forms of Cdh1 in human embryonic kidney 293T cells (HEK293T) confirmed decreased Cdh1 protein levels and revealed the inactivation of APC/C in the mutant form, leading to a decrease in the G0/G1 phase and increased in the S phase populations. Moreover, mutant Cdh1 failed to prevent the enlargement of the S phase caused by knockout of endogenous Cdh1 in cortical progenitor cells in primary culture. Our results indicate that APC/C‐Cdh1 loss of function caused by the Asp187Gly mutation impairs APC/C activity and underlies a new cause of prenatal microcephaly, psychomotor retardation, and epilepsy.

## Material and methods

### Identification of mutations in the *Fzr1* gene

We reviewed 390 studies by whole exome sequencing *in trio*, performed in our department since 2014, looking for mutations in the *Fzr1* gene. All studies were performed in patients suffering from neurodevelopmental disorders of probable genetic origin. Screened patients (117 females, 273 males) ranged in age from 5 months to 16 years. We identified a *de novo* missense heterozygous mutation in the *Fzr1* gene (hg19; chr 19: 3527718; NM_001136198.1; c.560A>G) that generates an aspartate‐to‐glycine substitution (p.Asp187Gly) in a 4‐year‐old boy from non‐consanguineous parents of Spanish origin. This mutation was typified as deleterious or pathogenic using the *in silico* prediction algorithms Protein variation effect analyzer (PROVEAN; Research Resource Identifier, RRID:SCR_002182), SIFT (RRID:SCR_012813), Polymorphism phenotyping‐2, MutationTaster (RRID:SCR_010777), and likelihood ratio test (Seifi and Walter 2018). The mutation was confirmed by Sanger sequencing.

### Clinical features of the patient

The *de novo* mutation was identified in a 4‐year‐old male patient from non‐consanguineous parents of Spanish origin. He was the product of a full‐term, 41‐week gestational pregnancy, via uncomplicated vaginal delivery to a 30‐year‐old primigravida mother. At 28th week of gestation ultrasound revealed microcephaly and intrauterine growth retardation. Apgar scores were 6 and 8 at 1 and 5 min, respectively. Patient body weight, height, and occipitofrontal diameter (OFD) are summarized in Table 1.

**Table 1:** Weight, height, and occipitofrontal diameter (OFD) values of the patient

Age	Weight (kg)	Height (cm)	OFD (cm)
At birth	2.5 (< 3rd centile)	49.5 (35th centile)	31 (< 3rd centile)
6 months	6.4 (< 3rd centile)	62 (5th centile)	41.5 (3rd centile)
8 months	7.8 (10th centile)	66 (10th centile)	43 (5th centile)
4 years	13.5 (< 3rd centile)	102 (10th centile)	47 (< 3rd centile)

Routine laboratory tests performed to the patient were: amino acids and organic acids in blood and urine; glucose, lactic and pyruvic acids, ammonium, pH and ketone bodies in blood; lactic and pyruvic acids, amines and their metabolites in cerebrospinal fluid.

The patient received the following antiepileptic therapy: valproic acid, rufinamide, levetiracetam, clonazepam, vigabatrine, topiramate, and ketogenic diet. Seizures persisted despite different combinations were utilized.

The study was carried out in accordance with the Declaration of Helsinki of the World Medical Association (2008) and approved by the Local Ethics Committees (Madrid, Spain; Ref. 30062019). Informed consent was obtained from parents, after full explanation of the procedures.

### Genetic study

Exome sequencing was performed using genomic DNA isolated (MagnaPure, 03003990001; Roche Diagnostics, Heidelberg, Germany) from whole blood from proband and parents. Blood samples were obtained at 08:30–09:30 hours. Libraries were prepared using the Ion AmpliSeq™ Exome Kit (A38264; Life Technologies, Grand Island, NY, USA, Thermo Fisher Scientific, Offenbach, Germany) and quantified by qPCR. The enriched libraries were prepared using Ion Chef™ and sequenced on PI™ Chip in the Ion Proton™ System (Life Technologies) to provide > 90% of amplicons covered with at least 20×. Signal processing, base calling, alignment, and variant calling were performed on a Proton™ Torrent Server using the Torrent Suite™ Software (A32199; Thermo Fisher Scientific). Variants were annotated using Ion Reporter™ Software (4487118; Thermo Fisher Scientific), and pedigree analysis was performed using the Genetic Disease Screen trio workflow. Variant filtering and prioritization were performed with an in‐house software program and a local database. Candidate variants were visualized using Integrative Genomics Viewer. Candidate variants were evaluated based on stringent assessments at both the gene and variant levels, taking into consideration both the patient's phenotype and the inheritance pattern. Variants were classified following the guidelines of the American College of Medical Genetics and Genomics (Cooley *et al.* 2013). A board of molecular clinical geneticists evaluated each variant classified as pathogenic, likely pathogenic, or a variant of uncertain significance, and decided which, if any, had to be reported. In every case, causal variants were discussed with the referring physician and/or clinical geneticist. Identified variants were confirmed by Sanger sequencing.

### Leucocyte extraction

Blood samples were obtained from patient and parents, at 08:30–09:30 hours. Leucocytes were isolated from whole blood by lysis of erythrocytes with ammonium chloride‐based lysing solution. Cells were washed and centrifuged (800 *g* for 5 min) with phosphate‐buffered saline (PBS) to remove non‐nucleated cells. Leucocyte viability was assessed by examination of trypan blue‐stained cells. Cell concentration was adjusted to 5 × 10^5^ cells/µL by resuspending the pellet with appropriate buffer for protein or RNA analysis.

### Cell culture and transfections

Human embryonic kidney 293T (HEK293T, ATCC® CRL‐3216™, RRID:CVCL_0063); the cell line is not listed as a commonly misidentified cell line by the International Cell Line Authentication Committee) cells were maintained in Dulbecco's modified Eagle's medium (D5546; Sigma‐Aldrich, St. Louis, MO, USA) supplemented with 10% (vol/vol) fetal calf serum (10270; Roche Diagnostics, Heidelberg, Germany). No further authentication was performed in the laboratory. A maximum of 15 cell passages was used. Twenty‐four hours before experiments, cells were seeded at 1.5 × 10^5^ cells/cm^2^ in plates previously coated with poly‐d‐lysine (15 µg/mL; p6407; Sigma‐Aldrich).

Primary cultures of cortical cells were prepared from wild‐type (Cdh1^+/+^) and Cdh1‐deficient (Cdh1^−/−^) (Delgado‐Esteban *et al.* 2013) mouse embryo (E14.5) cortices. Animals (C57BL/6J, JAX:000664; The Jackson Laboratories, Bar Harbor, ME USA; RRID:IMSR_JAX:000664) were maintained in specific pathogen‐free facilities at the University of Salamanca, in accordance with Spanish legislation (RD53/2013) under license from the Spanish government and the European Union (2010/63/EU). Protocols were approved by the Bioethics Committee of the Institute of Biomedical Research of Salamanca (Ref PI24/03/2018). Animals were bred at the Animal Experimentation Facility of the University of Salamanca in cages (maximum five animals/cage), and a light‐dark cycle was maintained for 12 h. Humidity was 45–65% and temperature 20–25°C. Animals were fed *ad libitum* with a standard solid diet (17% proteins, 3% lipids, 58.7% carbohydrates component, 4.3% cellulose, 5% minerals and 12% humidity) and free access to water. Animals (three pregnant mice) were euthanized using cervical dislocation. One embryo mouse was used for one cell culture. Cells were seeded at 2.0 × 10^5^ cells/cm^2^ in Dulbecco's modified Eagle's medium supplemented with 10% (vol/vol) fetal calf serum and incubated at 37°C in a humidified 5% CO_2_‐containing atmosphere. At 4 h after plating, cells were transfected with plasmid constructions. Experiments were performed at 1 day in culture, when cells are still considered neural progenitors (Delgado‐Esteban *et al.* 2013).

Plasmid transfection was performed using Lipofectamine® LTX (15338‐100; Invitrogen, Madrid, Spain), according to the manufacturer's instructions. We used the following plasmid constructions: (i) pIRES2‐EGFP (V35120; Invitrogen), either empty (expressing green fluorescent protein, GFP) or containing the full‐length cDNA of human Cdh1 (expressing both Cdh1 and GFP) (Maestre *et al.* 2008) and (ii) pcDNA™ 3.1(+) (V79020; Invitrogen), either empty or containing the full‐length cDNA of human Cdh1 fused to hemagglutinin (expressing hemagglutinin, HA‐Cdh1) (Bobo‐Jimenez *et al.* 2017). Cdh1 and HA‐Cdh1 were subjected to site‐directed mutagenesis on Asp187, which was replaced by Gly residue to obtain the mutated (Cdh1mut and HA‐Cdh1mut) form of Cdh1 (GenBank accession number NM_001136198.1), using the Quik Change XL kit (200524; Stratagene, La Jolla, CA, USA), followed by DpnI digestion. The forward and reverse oligonucleotides designed were 5'‐CCGAGCTGCAGGACGGCTTCTACCTCAATCT‐3' and 5'‐AGATTGAGGTAGAAGCCGTCCTGCAGCTCGG‐3', respectively. GFP‐transfected cells were identified by fluorescence microscopy and flow cytometry. Plasmid constructions will be shared upon reasonable request.

Bromodeoxyuridine (BrdU) incorporation into DNA and cell cycle phase percentage were determined by flow cytometry. This was achieved after 3h of incubation with 10 mg/mL BrdU using the APC BrdU Flow Kit (552598; BD Biosciences, San Jose, CA, USA), following the manufacturer's instructions (Delgado‐Esteban *et al.* 2013).

### Quantitative reverse transcription polymerase chain reaction (RT‐qPCR) analysis

Total RNA samples were purified from cells using a commercially available kit (RTN‐350; Sigma, St Louis, MO, USA) and RT‐qPCR was performed with Power SYBR Green RNA‐to‐CT TM 1‐Step kit (4389986; Applied Biosystems, Waltham, MA, USA). RT was carried out at 48°C for 30 min, and PCR conditions were 10 min at 95°C followed by 40 cycles of 15 s at 95°C and 1 min at 60°C using the appropriate forward and reverse primers, respectively (Sigma Aldrich): 5'‐AAGTCTCCCAGTCAGAACCG‐3' and 5'‐GTCCTGCACCTTCTCGATG‐3' (*Fzr1*, 0.2 µM; 5'‐TCAGCAATGCCTCCTGCACCA‐3' and 5'‐GCATGGACTGTGGTCATGAG‐3' (*GAPDH*, 0.3 µM). The mRNA levels of each transcript were normalized to the GAPDH mRNA abundance obtained from the same sample. The relative mRNA levels were calculated using the ΔΔCt method and were expressed as the fold change between sample and calibrator (Rodriguez *et al.* 2018).

### Western blot analysis

Cells were lysed in buffer containing 1% (vol/vol) nonidet NP‐40, 5 mM EDTA di‐K^+^, 20 mM Tris‐HCl pH 8.0, 137 mM NaCl, 10% (vol/vol) glycerol supplemented with phosphatase and protease inhibitors cocktail, stored on ice for 30 min and centrifuged at 13000 *g* for 5 min. Supernatants were collected and kept at −80°C until its use. Protein concentrations were determined with the bicinchoninic acid method, using bovine serum albumin as a standard (bicinchoninic acid Protein Assay kit, 23225; Thermo Fisher Scientific). Cell extracts were subjected to sodium dodecyl sulfate–polyacrylamide gel electrophoresis (10% sodium dodecyl sulfate; 8% Acrylamide : bis‐acriyamide 29 : 1; MiniProtean; Bio‐Rad Laboratories, Hercules, CA, USA). The resolved proteins were transferred electrophoretically to nitrocellulose membranes (Amersham™ Protram™, 10600018; Sigma‐Aldrich). Membranes were blocked with 5% (wt/vol) low fat milk in 20 mM Tris, 150 NaCl and 0.1% (vol/vol) Tween20, pH 7.5 for 1 h. After blocking, membranes were immunoblotted with primary antibodies overnight at 4°C. The antibodies used were anti‐Cdh1 (1 : 500; MS‐1116‐P1; Lab Vision, Thermo Fisher Scientific; RRID:AB_64167), anti‐HA (1 : 2000; 26183; Thermo Scientific; RRID:AB_10978021), anti‐Cyclin B1 (1 : 500; V152; Abcam, Cambridge, UK; RRID:AB_305751), anti‐Pfkfb3 (1 : 500; H00005209; Novus Biologicals, Centennial, CO, USA; RRID:AB_1112017), anti‐GFP; 1 : 2000; ab290; Abcam; RRID:AB_303395), and anti‐GAPDH (glyceraldehyde‐3‐phosphate dehydrogenase; 1 : 40000; AM4300; Thermo Fisher Scientific; RRID:AB_2536381). GAPDH was used as loading control. After incubation with horseradish peroxidase‐conjugated mouse anti‐rabbit IgG (sc‐2357; Santa Cruz Biotechnology, Dallas, TX, USA; RRID:AB_628497) or goat anti‐mouse IgG (172‐1011, Bio‐Rad, RRID:AB_11125936) for 1 h at 22ºC, membranes were incubated with the enhanced chemiluminescence Pierce™ enhanced chemiluminescence Plus Western Blotting Substrate (32132, Thermo Fisher Scientific) for 5 min, before exposure to Kodak XAR‐5 film, and the autoradiograms were scanned. Band intensities were quantified using ImageJ1.48v software (NIH, Bethesda, MD, USA) (Rodriguez *et al.* 2017).

### Immunocytochemistry

Cells grown on glass coverslips were fixed with 4% (wt/vol, in PBS) paraformaldehyde for 30 min and subsequently incubated in permeabilization buffer (0.25%. triton in PBS, vol/vol) for 5 min, and blocking buffer (0.1% Triton, 10% goat serum in PBS, vol/vol) for 1 h. Afterward, cells were immunostained with rabbit anti‐HA (1 : 500; 3724 Cell Signaling Technology, Beverly, MA, USA; RRID:AB_1549585) and immunolabeling was detected using IgG‐Cy3 secondary antibody (1 : 500; 111‐165‐003; Jackson ImmunoResearch Labs, Cambridge, UK; RRID:AB_2338000). Nuclei were stained with 6‐diamidino‐2‐phenylindole (D9542; Sigma‐Aldrich). Coverslips were washed, mounted with SlowFace light antifade reagent (S2828; Invitrogen) and examined under a microscope (Nikon Inverted microscope Eclipse Ti‐E; Nikon, Tokyo, Japan) or a scanning laser confocal microscope (Olympus IX81 Spinning disk confocal microscope; Olympus, Tokyo, Japan).

### Statistical analysis

All measurements were carried out at least in triplicate, and the results are expressed as mean ± SEM. Normality of data was carried out by the Kolmogorov–Smirnov (K–S) test. A one‐way or two‐way anova with a least significant difference *post hoc* test was used to compare values between multiple groups, and a two‐tailed, unpaired Student's *t*‐test was used for two‐group comparisons. In all cases, *p* < 0.05 were considered significant. Statistical analyses were performed using spss Statistics 24.0 for Macintosh (IBM, Armonk, New York, NY, USA). No sample calculation was performed.

No data point was excluded from data analysis. No outliers were detected by graphical techniques.

This study was not pre‐registered.

During immunocytochemistry and flow cytometry studies, the investigators were blinded to experimental group (Cdh1 and Cdh1mut cell transfections). Cell cultures were identified by numbers, which were informed to the investigator only after finishing experiments.

## Results

### A *de novo* human *Fzr1* missense mutation results in prenatal microcephaly, psychomotor retardation, and epilepsy

We previously found that genetic ablation of *Fzr1* promotes the death of neural progenitor cells leading to neurogenesis impairment and microcephaly in mouse. However, no Fzr1 variant has so far been reported to cause any disease phenotype in humans. To ascertain the possible translation of our findings in humans, here we searched for mutations in the *Fzr1* gene in 390 whole exomes sequenced *in trio* in individuals showing neurodevelopmental disorders compatible with a genetic origin. We found a *de novo* missense mutation in the *Fzr1* gene (hg19; chr 19: 3527718; NM_001136198.1; c.560A>G) that generates an aspartate‐to‐glycine substitution (p.Asp187Gly) in a patient presenting microcephaly at birth, as revealed by the low OFD (< 3rd centile), and low body weight (< 3rd centile) (Table 1). In the days following birth, low weight, dyspnea, and right hemiclonic seizures were recorded. The frequency of seizures increased gradually over the next 3–4 months to several episodes per day. The seizures were refractory to improvement with antiepileptic drugs in mono or polytherapy. At the age of 4 months, the patient showed right and left hemiclonic seizures, left focal seizures, generalized tonic‐clonic seizures, and versive seizures. A video‐electroencephalogram evaluation showed numerous polymorphic focal seizures; the corresponding ictal electroencephalogram showed seizure origin changing from left hemisphere and from right hemisphere, showing virtually continuous migratory ictal foci. Routine laboratory screening including thyroid function and neurometabolic tests were within the normal range. Brain 3‐Tesla MRI did not reveal any significant abnormalities. Cardiac ultrasound study showed a mild mitral and tricuspid insufficiency and left ventricular hypertrophy.

At 6 months of age, physical examination showed a failure to thrive with body weight and OFD less than 3rd centile and at 3rd centile, respectively, and height at the 5th centile. At 4‐year‐old, body weight and OFD remained less than 3rd centile (Table 1). At the age of 8 months, the patient was evaluated because of severe psychomotor retardation and refractory seizures (around 10–30 seizures per day). At that age, his weight, height, and OFD were 7.8 kg (10th centile), 66 cm (10th centile), and 43 cm (5th centile), respectively (Table 1). Physical examination revealed severe axial hypotonia, exaggerated deep‐tendon reflexes, and spasticity. Finally, at the age of 4 years, the patient does not walk, does not say words, and his contact with the environment is scarce.

Hence, we have identified a *de novo* mutation in the human *Fzr1* gene that results in prenatal microcephaly, refractory epilepsy, and psychomotor retardation.

### Asp187Gly mutation in Cdh1 results in decreased protein levels and APC/C activity

**Figure 1:**
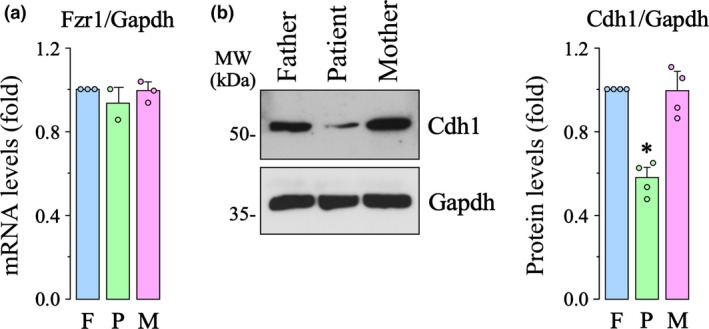
Patient carrying the 560A>G mutation in the Fizzy‐related protein 1 (*Fzr1*) gene expresses lower Cdh1 protein levels. Whole leucocyte lysates were obtained from patient (P) and parental blood and protein and RNA were extracted. Father (F) and mother (M) do not carry the mutation and was considered as controls. (a) Isolated total RNA was subjected to RT‐qPCR analysis. The Fzr1 mRNA abundance was normalized to the GAPDH mRNA levels obtained in each same sample. The relative mRNA levels were considered as the fold change in relation to father value. Fzr1 RNA levels were similar in leucocyte extracts from patient and his parents. (b) Representative western blot images showing Cdh1 protein levels in patient, mother and father leucocyte protein extracts. Quantification of protein levels shows decreased Cdh1 protein expression in the patient carrying the mutation, in comparison to levels detected in both father and mother cells. The relative protein levels were expressed as the fold change in relation to the father value. GAPDH was used as loading control. Values are mean ± SEM from 2 to 4 different leucocyte extracts. *p < 0.05 compared with father values.

**Figure 2:**
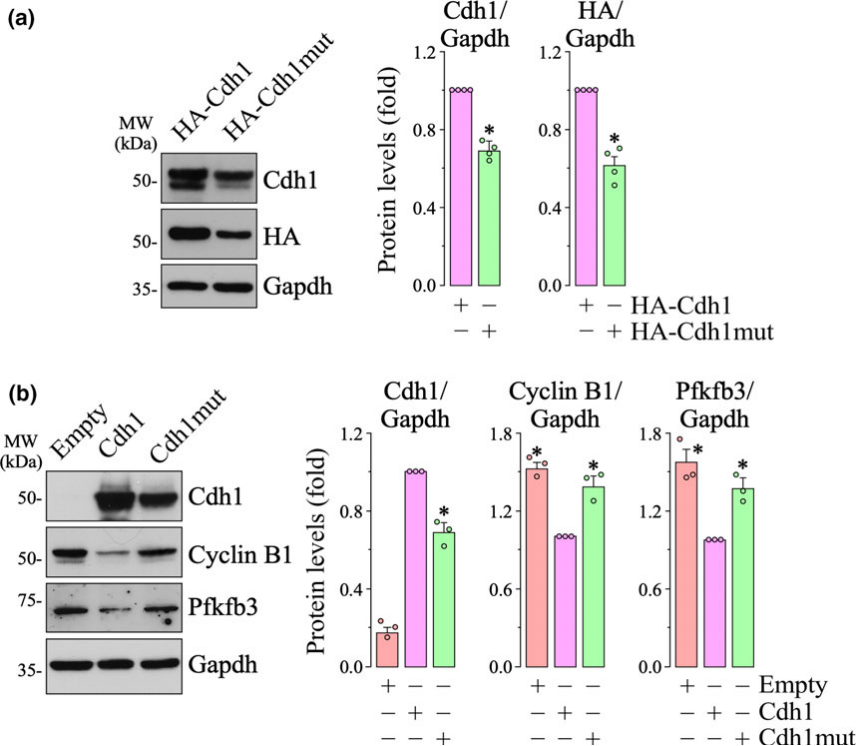
Human cells expressing the Asp187Gly mutant form of Cdh1 results in decreased protein levels and anaphase‐promoting complex/cyclosome (APC/C) activity. (a) Human embryonic kidney 293T cells (HEK293T) cells were transfected with hemagglutinin (HA)‐tagged mammalian expression vector encoding either wild‐type (HA‐Cdh1) or mutant (HA‐Cdh1mut) forms of Cdh1. Western blot analysis shows decreased Cdh1 and HA protein levels in the mutant form. (b) HEK293T cells were transfected with the empty expression vector (Empty) and plasmids encoding either wild‐type (Cdh1) or mutant (Cdh1mut) forms of Cdh1. Western blot analysis shows decreased Cdh1 protein levels and lower APC/C activity, as revealed by the accumulation of its targets, cyclin B1 and Pfkfb3. The relative protein levels were expressed as the fold change in relation to the wild‐type Cdh1 value. GAPDH was used as a loading control. Values are mean ± SEM from 3 to 4 different cell transfections. *p < 0.05 compared with (a) HA‐Cdh1 or (b) Cdh1.

Next, we evaluated the functional relevance of the Cdh1 Asp187Gly mutant form. Cdh1 is expressed broadly (Gieffers *et al.* 1999), including blood cells (Colombo *et al.* 2010). To study the effect of patient Asp187Gly mutation, we first isolated whole leucocyte extracts from patient and parental blood and Cdh1 level expression was analyzed. Parents samples were used as control, as both the father and the mother did not carry the mutation. As shown in Fig. 1(a), mRNA levels were similar in the cell extracts from the patient and his parents, suggesting that the mutation does not affect the rate of *Fzr1* gene expression or mRNA stability. However, western blot analysis revealed decreased Cdh1 protein abundance in the patient when compared with the father or the mother (Fig. 1b). To confirm this finding, we next introduced the Asp187Gly mutation into Cdh1 by site‐directed mutagenesis into a HA‐tagged mammalian expression vector. Transfection of HEK293T cells with identical amounts of either wild‐type or mutant Cdh1 cDNA resulted in a decreased Cdh1 protein expression in the mutant form, as revealed by both HA and Cdh1 immunodetection (Fig. 2a). These results are compatible with the notion that the Cdh1 Asp187Gly mutation impairs Cdh1 protein stability.

Next, we tested whether the identified Cdh1 mutation affects APC/C activity. To avoid the possible interference of the HA tag on Cdh1 function, we transfected HEK293T cells with either the wild‐type or the Asp187Gly mutant forms of untagged Cdh1. The results confirmed the decreased protein expression of the mutant form (Fig. 2b). Moreover, APC/C activity was found to be lower in cells expressing the Cdh1 mutant form, as revealed by the accumulation of the well‐known APC/C targets, cyclin B1 (Irniger *et al.* 1995; Thornton and Toczyski 2003) and Pfkfb3 (Herrero‐Mendez *et al.* 2009), when compared with cells expressing wild‐type Cdh1 (Fig. 2b).

**Figure 3:**
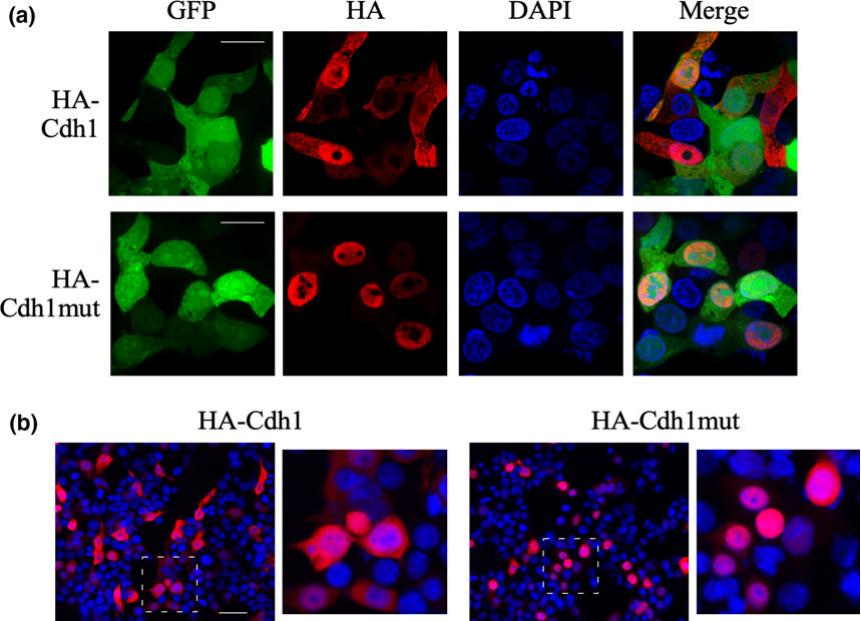
The Asp187Gly mutant form of Cdh1 is confined to the nucleus. (a) human embryonic kidney 293T cells (HEK293T) cells were co‐transfected with mammalian expression vector, encoding either hemagglutinin (HA)‐tagged wild‐type (HA‐Cdh1) or mutant (HA‐Cdh1mut) forms of Cdh1, together with plasmids encoding the green florescent protein (GFP). Immunostaining revealed that the mutant (HA‐Cdh1mut) is confined to the nuclei, whereas wild‐type Cdh1 localizes in both the nucleus and cytosol. Bar: 15 µm. (b) HEK293T cells were transfected with mammalian expression vectors expressing either HA‐tagged wild‐type (HA‐Cdh1) or mutant forms (HA‐Cdh1mut) of Cdh1, in the absence of GFP co‐expression. Immunostaining confirmed the nuclear accumulation of the mutant form of Cdh1. Bar: 50 µm.

**Figure 4:**
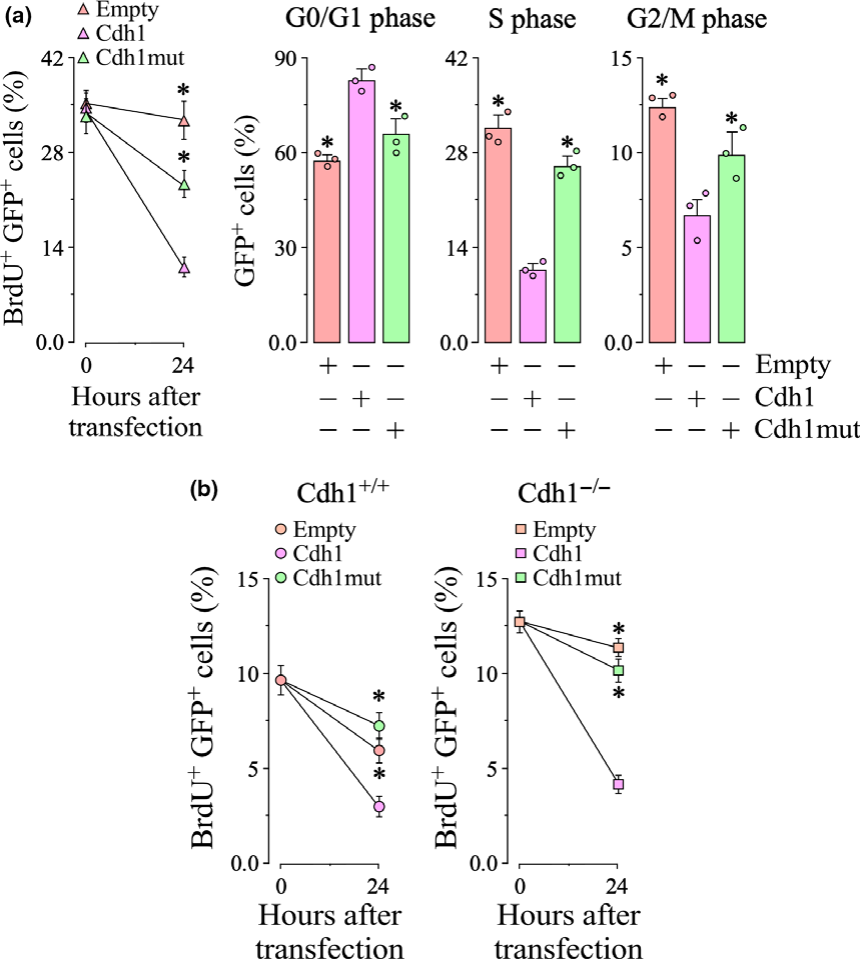
The Asp187Gly mutant form of Cdh1 increases S phase length in human cell line and primary cultured cortical cells. (a) Human embryonic kidney 293T cells cells were transfected with mammalian expression vectors co‐expressing green fluorescent protein (GFP) and either wild‐type (Cdh1) or mutant forms (Cdh1mut) of Cdh1. Empty vector only express GFP. Bromodeoxyuridine (BrdU) incorporation was measured by flow cytometry analysis in the GFP^+^ (transfected cells) populations. At 24 h after transfections, cells expressing Cdh1mut maintained their ability to incorporate BrdU and cell cycle activation, as showed by the decrease in the G0/G1 phase and increase in the S and G2/M phases. Values are mean ± SEM from three different cell transfections. (a) Primary cortical cells were cultured from wild‐type (Cdh1^+/+^) and knockout (Cdh1^−/−^) Cdh1 mice. At 4 h of culture, cells were transfected with mammalian expression vectors co‐expressing GFP and either wild‐type (Cdh1) or mutant forms (Cdh1mut) of Cdh1. At 24 h after transfections, Cdh1 expression decrease BrdU incorporation in both transfected (GFP^+^) Cdh1^+/+^ and Cdh1^−/−^ cortical cells. In contrast, Cdh1^−/−^ progenitors expressing Cdh1mut maintained their ability to incorporate BrdU. Values are mean ± SEM from three different cortical cell cultures. *p < 0.05 compared with Cdh1.

The subcellular localization of Cdh1 provides a key element of spatial regulation of APC/C‐Cdh1 activity. Cdh1 phosphorylation induces its nuclear export to the cytosol, contributing to the efficient inactivation of APC/C‐Cdh1 (Jaquenoud *et al.* 2002; Maestre *et al.* 2008). To ascertain whether the Cdh1 Asp187Gly mutation affects Cdh1 subcellular localization, we next performed immunostaining of the cells transfected with either the wild‐type or mutant Cdh1. The results revealed that both wild‐type (HA‐Cdh1) and mutant (HA‐Cdh1mut) Cdh1 were present in the nucleus (Fig. 3a), i.e. accessible to Cdh1 cell cycle‐protein targets, although with different localization pattern. Whereas the wild‐type Cdh1 also spread to the cytosol, the mutant form was confined within the nucleus (Fig. 3a). However, analysis of the cell cycle distribution revealed differences in cells expressing wild‐type versus those expressing the mutant Cdh1. Thus, expression of wild‐type Cdh1 decreased BrdU incorporation and promoted cell cycle arrest, as judged by the increased G0/G1 phase and the decreased S and G2/M phases (Fig. 4a). In contrast, cells expressing the mutant Cdh1 maintained their ability to incorporate BrdU and cell cycle activation, as showed by the decrease in the G0/G1 phase and increase in the S and G2/M phases (Fig. 4a). These data suggest that the Cdh1 Asp187Gly mutation fails to arrest cell cycle and triggers shortening of the G1 phase and enlargement of the S phase.

Previously, we have reported that genetic elimination of Cdh1 prolongs S phase in neural progenitor cells, which delays the cell cycle exit and the onset of neurogenesis (Delgado‐Esteban *et al.* 2013). To assess the possible influence of the Cdh1 Asp187Gly mutation in neurogenesis, which could explain the microcephaly observed in the patient, we studied the cell cycle distribution in primary cortical progenitor cells obtained from either wild‐type (Cdh1^+/+^) or knockout (Cdh1^−/−^) mice (Delgado‐Esteban *et al.* 2013). Flow cytometry analysis showed that BrdU incorporation decreased in Cdh1^+/+^, but not in Cdh1^−/−^ progenitor cells, which maintained their ability to incorporate BrdU as we previously found (Delgado‐Esteban *et al.* 2013). However, in contrast to the wild‐type Cdh1, expression of the Asp187Gly mutant failed to decrease BrdU incorporation in Cdh1^−/−^ cortical progenitors (Fig. 4b), further confirming the inactivation of APC/C activity.

Together, our results indicate that the Cdh1 Asp187Gly mutation impairs APC/C activity, as revealed by the accumulation of Cdh1 protein‐targets Cyclin B1 and Pfkfb3, and the aberrant cell cycle distribution in replicative cells. Moreover, the Cdh1 mutant triggers S phase enlargement in cortical progenitor cells, which might result in replicative stress and apoptotic death of neural precursor cells (Delgado‐Esteban *et al.* 2013). These results are compatible with prenatal microcephaly found in the patient carrying the *de novo* mutation in the human *Fzr1* gene herein identified.

## Discussion

Here, we describe a *de novo* missense mutation (c.560A>G) in the human *Fzr1* gene that predicts an aspartate‐to‐glycine substitution (p.Asp187Gly) in the Cdh1 protein, which results in prenatal microcephaly, psychomotor retardation, and severe epilepsy. We found decreased Cdh1 protein abundance in the patient cells. Moreover, the Asp187Gly mutation may cause APC/C‐Cdh1 loss of function, as revealed by the accumulation of protein targets, Cyclin B1 and Pfkfb3, and observed aberrant cell cycle distribution in human proliferative cells. Furthermore, the Asp187Gly mutant fails to induce cell cycle arrest in Cdh1^−/−^ cultured cortical cells, leading to increased BrdU incorporation (S phase). These findings indicate that APC/C‐Cdh1 loss of function induced by the Asp187Gly mutation results in a new cause of prenatal microcephaly, psychomotor retardation, and severe epilepsy.

We previously described that APC/C‐Cdh1 coordinates cortical neurogenesis and size during development (Delgado‐Esteban *et al.* 2013). The loss of Cdh1 reduces the length of the G1 phase and increases S‐phase duration in neural precursor cells, which generates replicative stress and apoptotic death of neural precursor cells, leading to microcephaly (Delgado‐Esteban *et al.* 2013; Eguren *et al.* 2013). In the patient harboring the genetic 560A>G mutation, Cdh1 protein abundance was lower, which is compatible with decreased APC/C activity. In fact, APC/C activity has been shown to be regulated by Cdh1 protein abundance (Kramer *et al.* 2000; Listovsky *et al.* 2004; Nagai *et al.* 2018). Our data showing the accumulation of Cdh1 protein targets, namely Cyclin B1 and Pfkfb3, and aberrant cell cycle distribution, in human proliferative cells, confirms the loss of function APC/C‐Cdh1 in the Asp187Gly mutants. Moreover, expression of the Cdh1 Asp187Gly mutant in Cdh1^−/−^ cultured cortical cells failed to induce cell cycle arrest, as reveals the enlargement of the S phase length, a feature known to result in prenatal microcephaly (Delgado‐Esteban *et al.* 2013; Eguren *et al.* 2013) as observed in the patient harboring the 560A>G missense mutation.

Whereas the Cdh1 Asp187Gly mutation does not affect mRNA expression, it results in lesser Cdh1 protein abundance in both the patient and in the human proliferating cell line, suggesting that this mutation affects protein stability. APC/C‐Cdh1 function is cell cycle‐regulated and controlled by the rates of Cdh1 synthesis and destruction (Kramer *et al.* 2000). In fact, Cdh1 levels are low during late G1 phase and S phase and increase as cells enter mitosis (Kramer *et al.* 2000; Nagai *et al.* 2018). Moreover, Cdh1 instability is promoted by forcing the accumulation of Cdh1 into the nucleus (Nagai *et al.* 2018), where it is targeted for degradation by E3 ubiquitin ligases, APC/C (Listovsky *et al.* 2004; Nagai *et al.* 2018) and Skp1 Cullin1 F‐box (SCF) (Benmaamar and Pagano 2005; Fukushima *et al.* 2013; Nagai *et al.* 2018). Interestingly, we found that, in contrast to wild‐type Cdh1, the localization of the mutant form is confined to the nucleus, hence being more exposed to ubiquitination likely explaining its protein instability.

It has been reported that the *FZR1* gene has a low haploinsufficiency (Lek *et al.* 2016). The intolerance score is 1 and the haploinsufficiency score is 42 (Firth *et al.* 2009). Both scores, but particularly the first one, point out that a loss of function mutation in one allele will hardly be compensated by the non‐mutated allele. According to the intolerance score, we should assume that mutations of this gene in heterozygosis should have a significant impact on Cdh1 protein levels and on the development of the central nervous system, as we herein identified.

Notably, the patient that we identified harbors the 560A>G mutation, shows partial migratory seizures, that is, a rare and severe early infantile epileptic encephalopathy (Coppola *et al.* 1995) with an estimated prevalence of 0.11 per 100 000, and an overall incidence rate of 0.26–0.55 cases per 100 000 live births (McTague *et al.* 2013). The main features of the migratory seizures are the presence of normal development before the onset of epilepsy, the onset of seizures before 6 months, the migration of epileptic discharges, refractory seizures, and important psychomotor retardation (McTague *et al.* 2013; Striano *et al.* 2014). The underlying mechanisms of the disease are poorly understood, and a limited number of genetic causes have been described (Coppola *et al.* 1995; Carranza Rojo *et al.* 2011; Barcia *et al.* 2012; Striano *et al.* 2014). The role of the *Fzr1* gene, as well as the existence of antenatal microcephaly, broadens the genotype and phenotype of this epileptic encephalopathy, providing added value to our findings. In this context, APC/‐Cdh1 regulates transmission at glutamatergic synapses (Juo and Kaplan 2004; Fu *et al.* 2011; Huang *et al.* 2015) and γ‐aminobutyric signaling (Kowalski *et al.* 2014). Thus, the loss of Cdh1 may alter the excitatory to inhibitory balance, hence explaining the occurrence of epilepsy in the patient herein identified.

Finally, the fact that this mutation is *de‐novo*, and no allele frequency is reported in healthy individuals makes it more likely to cause the patient's phenotype. Interestingly, it involves the first conserved WD‐domain of the protein, known to be important for protein‐protein interactions (Watson *et al.* 2019). Thus, the relevance of the 560A>G mutation on Cdh1 interaction with its targets might be evaluated in the future.

In conclusion, here we describe a new pathology characterized by antenatal microcephaly, psychomotor retardation, and severe epilepsy, secondary to heterozygous mutations of the *Fzr1* gene. This case points out the potential of exome sequencing for the typing of new syndromes, as well as the implication of hitherto unknown genes in refractory epilepsies.

### Open science badges

This article has received a badge for *Open Materials* because it provided all relevant information to reproduce the study in the manuscript. The complete Open Science Disclosure form for this article can be found at the end of the article. More information about the Open Practices badges can be found at https://cos.io/our-services/open-science-badges/.
